# Variation in Estimated Ozone-Related Health Impacts of Climate Change due to Modeling Choices and Assumptions

**DOI:** 10.1289/ehp.1104271

**Published:** 2012-07-12

**Authors:** Ellen S. Post, Anne Grambsch, Chris Weaver, Philip Morefield, Jin Huang, Lai-Yung Leung, Christopher G. Nolte, Peter Adams, Xin-Zhong Liang, Jin-Hong Zhu, Hardee Mahoney

**Affiliations:** 1 Environment and Resources Division, Abt Associates Inc., Bethesda, Maryland, USA; 2 Global Change Research Program, National Center for Environmental Assessment, Office of Research and Development, U.S. Environmental Protection Agency, Washington, DC, USA; 3 Pacific Northwest National Labs, Richland, Washington, USA; 4 National Exposure Research Laboratory, U.S. Environmental Protection Agency, Athens, Georgia, USA; 5 Civil and Environmental Engineering, Carnegie Mellon University, Pittsburgh, Pennsylvania, USA; 6 Department of Atmospheric Sciences, University of Illinois, Urbana, Illinois, USA

**Keywords:** climate change, mortality, ozone, population projections, sensitivity analysis

## Abstract

Background: Future climate change may cause air quality degradation via climate-induced changes in meteorology, atmospheric chemistry, and emissions into the air. Few studies have explicitly modeled the potential relationships between climate change, air quality, and human health, and fewer still have investigated the sensitivity of estimates to the underlying modeling choices.

Objectives: Our goal was to assess the sensitivity of estimated ozone-related human health impacts of climate change to key modeling choices.

Methods: Our analysis included seven modeling systems in which a climate change model is linked to an air quality model, five population projections, and multiple concentration–response functions. Using the U.S. Environmental Protection Agency’s (EPA’s) Environmental Benefits Mapping and Analysis Program (BenMAP), we estimated future ozone (O_3_)-related health effects in the United States attributable to simulated climate change between the years 2000 and approximately 2050, given each combination of modeling choices. Health effects and concentration–response functions were chosen to match those used in the U.S. EPA’s 2008 Regulatory Impact Analysis of the National Ambient Air Quality Standards for O_3_.

Results: Different combinations of methodological choices produced a range of estimates of national O_3_-related mortality from roughly 600 deaths avoided as a result of climate change to 2,500 deaths attributable to climate change (although the large majority produced increases in mortality). The choice of the climate change and the air quality model reflected the greatest source of uncertainty, with the other modeling choices having lesser but still substantial effects.

Conclusions: Our results highlight the need to use an ensemble approach, instead of relying on any one set of modeling choices, to assess the potential risks associated with O_3_-related human health effects resulting from climate change.

There is a substantial and growing literature on the potential impacts of climate change in the absence of efforts to mitigate the atmospheric accumulation of greenhouse gases due to global emissions and other factors. The recent Intergovernmental Panel on Climate Change (IPCC) Fourth Assessment Report found that “warming of the climate system is unequivocal” and that “most of the observed increase in globally averaged temperatures since the mid-20th century is very likely due to the observed increase in anthropogenic greenhouse gas concentrations” (IPCC 2007). Of particular importance for the U.S. Environmental Protection Agency’s (EPA’s) mission to protect human health and the environment is the potential for future climate change to cause air quality degradation via climate-induced changes in meteorology and atmospheric chemistry, which poses challenges to the U.S. air quality management system and the effectiveness of its pollution mitigation strategies (IPCC 2007; Isaksen et al. 2009; Jacob and Winner 2009; National Research Council 2004). In this context, the Global Change Research Program in the U.S. EPA’s Office of Research and Development, in partnership with its Office of Air and Radiation, began soliciting research that targeted the impacts of climate change on air quality in 1999 (U.S. EPA 2009a; Weaver et al. 2009).

To move from a consideration of environmental impacts to an explicit assessment of human health risks, the demographics, and the size of the exposed population, whether now or in the future, is a critical input to any analysis of the human health effects related to climate change. Therefore, the U.S. EPA has concurrently been developing high-resolution, spatially explicit population projections for the United States. These projections, from the Integrated Climate and Land-Use Scenarios (ICLUS) project (U.S. EPA 2009b), have been developed to be consistent with the underlying assumptions of the IPCC Special Report on Emissions Scenarios (SRES) social, economic, and demographic storylines (Nakicenovic et al. 2000; U.S. EPA 2009b).

Our work builds on these two efforts by examining the potential indirect impacts of climate change on the health of a hypothetical future U.S. population [in approximately (ca.) year 2050] via its direct impact on tropospheric ozone (O_3_) concentrations. We input both the results of the linked climate change and air quality models (hereafter referred to as the climate change–air quality modeling systems) and various population projections into the Environmental Benefits Mapping and Analysis Program (BenMAP), the U.S. EPA’s air pollution benefits analysis model, to estimate the changes in adverse health effects resulting from the changes in ambient O_3_ concentrations simulated by the climate change–air quality modeling systems. Our analysis considers the health impacts associated with O_3_ changes induced only by future climate change; the air quality modeling simulated the response of O_3_ to global climate change alone, without changes in anthropogenic emissions of O_3_ precursors [e.g., due to future air quality management efforts and future economic growth, as described previously (U.S. EPA 2009a; Weaver et al. 2009)].

In several studies, investigators modeled the health impacts of climate change–induced changes in O_3_ (Bell et al. 2007; Hwang et al. 2004; Knowlton et al. 2004; Sheffield et al. 2011; Tagaris et al. 2009; West et al. 2007). All of these studies found that simulated climate change produced increases in O_3_-related mortality. Tagaris et al. (2009) also found the potential for additional PM_2.5_-related mortality due to climate change. However, few studies have investigated the sensitivity of their estimates to the underlying modeling choices. For example, each of the references cited used a single climate change–air quality modeling system as the basis for their analysis, although Tagaris et al. (2009) did provide a useful estimate of the uncertainty surrounding their O_3_-related health findings based on the range of results reported in Weaver et al. (2009). Similarly, only West et al. (2007) considered population growth in their analysis. Therefore, instead of developing a quantitative estimate of future human health impacts of climate-induced O_3_ changes, our goal, building on these previous studies, was to assess the sensitivity of such estimates to key modeling assumptions and choices. Our purpose was to explore the uncertainty surrounding the assessment of these climate-related health impacts and to sketch out the set of health risks that society must begin to consider.

## Methods

Our study was designed to assess the sensitivity of projected future O_3_-related human health impacts in the United States to modeling and methodological choices for *a*) climate-induced changes in future meteorological conditions; *b*) the changes in O_3_ concentrations resulting from those meteorological changes; *c*) the size of the affected population, as well as its age and geographic distributions; and *d*) the concentration–response (C–R) relationships linking O_3_ levels to specific health outcomes.

**Figure 1:**
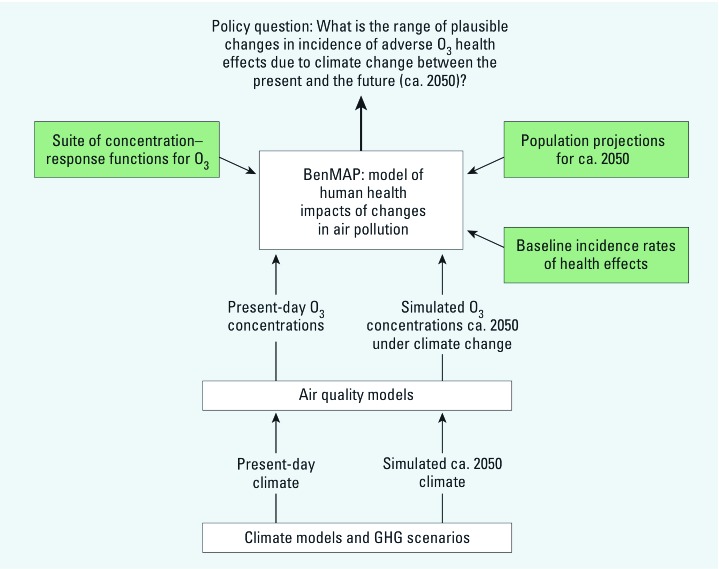
The structure of the analysis of O_3_-related impacts on human health attributable to climate change. GHG, greenhouse gas.

There is substantial uncertainty surrounding each of the inputs to our analysis, particularly because it focuses so far into the future. Much of this uncertainty cannot be assessed quantitatively. Even assigning probabilities to the different models (representing our subjective assessments about the relative accuracy with which each approximates a future reality) is premature. Instead, we present our analysis as a series of sensitivity analyses or “what if” scenarios designed to assess the impact of the various assumptions and modeling approaches on the results. Figure 1 illustrates the basic structure of the analysis.

*Climate change–air quality modeling systems.* Our analysis includes seven modeling efforts of six research groups: Harvard University; Carnegie Mellon University (CMU); Washington State University (WSU); U.S. EPA’s National Exposure Research Laboratory (NERL); the joint efforts of the Georgia Institute of Technology, the Northeast States for Coordinated Air Use Management, and the Massachusetts Institute of Technology (GNM); and the University of Illinois, which considered two different SRES scenarios (denoted Illinois-1 and Illinois-2), but otherwise used identical setups. The Harvard and CMU simulations used global-scale (e.g., 4° × 5° grids) atmospheric chemistry models. The remaining simulations used regional air quality models, which necessitates downscaling global climate model data to fine scales (e.g., 36-km grids). These modeling efforts are described in detail elsewhere (U.S. EPA 2009a; Weaver et al. 2009); we have summarized the key characteristics in Tables 1 and 2. Briefly, each modeling group explored the potential impacts of climate change on O_3_ concentrations in the United States using two linked models. First, we used a climate model to simulate meteorological conditions in the United States for future years (under climate change) and in the present. This modeled meteorology was then input to an air quality model to simulate the ambient O_3_ concentrations that would result. Anthropogenic emissions were held constant between the base case and the climate change case, but climate-sensitive biogenic and evaporative emissions were allowed to change in response to changes in climate. Baseline emissions were similar, although not identical, across modeling efforts (e.g., for the United States, based largely on 1999 or 2001 U.S. EPA emissions inventory data), as detailed in the references cited by the U.S. EPA (2009a) and Weaver et al. (2009). Some modeling groups used dynamical downscaling (with a regional climate model) to further regionalize the global climate model simulation outputs. The choice of downscaling model and methodology is an additional source of uncertainty, but systematically separating out this additional source was not feasible for this analysis.

**Table 1:** Summary of global climate and O_3_ modeling systems used in this analysis.

Modeling system	Harvard	CMU
Simulation period	5 summers/falls	10 summers/falls
GCM	GISS III	GISS II’
Resolution	4° × 5°	4° × 5°
GHG scenario	A1b	A2
GCTM	GEOS-chem	GISS II’
Climate sensitive emissions	BVOCs; lightning and soil NOx	BVOCs; lightning and soil NOx
Abbreviations: A1b, A2, the names given to the SRES scenarios of greenhouse-gas (GHG) emissions used to drive the climate models; BVOC, biogenic volatile organic compounds; GCM, general circulation model; GCTM, global chemical transport model; GEOS, Goddard Earth Observing System; GISS, Goddard Institute for Space Studies; NOx, nitrogen oxides.				

**Table 2:** Summary of regional climate and O_3_ modeling systems.

Modeling system	NERL	Illinois-1	Illinois-2	WSU	GNM
Simulation period	5 JJAs	4 JJAs	4 JJAs	5 Julys	3 JJAs
GCM	GISS III	PCM	PCM	PCM	GISS III
Global resolution	4° × 5°	2.8° × 2.8°	2.8° × 2.8°	2.8° × 2.8°	4° × 5°
GHG scenario	A1b	A1Fi	B1	A2	A1b
RCM	MM5	CMM5	CMM5	MM5	MM5
Regional resolution	36 km	90/30 km	90/30 km	36 km	36 km
Convection scheme	Grell	Grell	Grell	Kain–Fritsch	Grell
RAQM	CMAQ	AQM	AQM	CMAQ	CMAQ
Chemical mechanism	SAPRC99	RADM2	RADM2	SAPRC99	SAPRC99
Climate sensitive emissions	BVOCs; evaporative	BVOCs; evaporative	BVOCs; evaporative	BVOCs; evaporative	BVOCs; evaporative
Abbreviations: A2, A1b, A1Fi, B1, and B2, the names given to the SRES scenarios of greenhouse-gas (GHG) emissions used to drive the climate models; AQM, air quality model; BVOC, biogenic volatile organic compounds; CMAQ, Community Multiscale Air Quality Model; CMM5, University of Illinois climate extension of the Penn State/National Center for Atmospheric Research (NCAR) Mesoscale Model, version 5; GCM, general circulation model; GISS, Goddard Institute for Space Studies; JJA, June, July, August; Grell and Kain-Fritsch, convective parameterizations in the regional climate models; MM5, Penn State/NCAR Mesoscale Model, version 5; PCM, parallel climate model; RADM2, Regional Atmospheric Deposition Model (2nd generation); RAQM, regional air quality model; RCM, regional climate model; SAPRC, statewide air pollution research center; SAPRC99, one of the chemical mechanism packages used in the CMAQ model.										

The modeling groups produced from 3 to 10 summers of maximum daily 8-hr average ozone concentrations (MDA8) that were approximately centered on the years 2000 (present) and 2050 (future). The MDA8 was computed by taking rolling 8-hr averages for a 24-hr period and then taking their maximum. This was performed for all days in the modeled O_3_ seasons. Although different models used different grids, the air quality grids for all of the models were remapped to a 30 km × 30 km grid for this analysis for consistency. Further adjustment of modeled air quality is described in Supplemental Material, p. 3 (http://dx.doi.org/10.1289/ehp.1104271).

*Population projections to a future year.* All of the BenMAP runs used populations projected to 2050. To explore the sensitivity of our results to assumptions about what this future population would look like, we selected five population projections for input into our analysis. One of these was simply the 2000 Census population (i.e., we assumed no change from the 2000 Census population by 2050 to show the risk associated with climate change in the absence of changes in populations exposed) (U.S. EPA 2010b). A second population projection is extrapolated from the Woods & Poole population projections for the year 2030 already in BenMAP (Woods & Poole Economics Inc. 2007), using a set of exponential smoothing forecasting methods [for details, see Supplemental Material, pp. 3–4 (http://dx.doi.org/10.1289/ehp.1104271)]. Finally, we selected three of the ICLUS population projections—A1, A2, and the base case—to provide the lower and upper bound ICLUS total population projections, as well as an intermediate case. The basis for the ICLUS population projects and the underlying assumptions are described in detail elsewhere (U.S. EPA 2009b) and more briefly in the Supplemental Material, p. 4.

*C–R relationships and health impact functions.* We followed the selection of health effects, studies, and C–R functions that the U.S. EPA used in the benefits analysis for the Regulatory Impact Analysis of the National Ambient Air Quality Standards for O_3_, which was completed in 2008 (U.S. EPA 2008; 2010a). The C–R functions are taken from epidemiological studies, and we assumed they were applicable to any year, although this assumption entails additional uncertainties. The suite of health effects included mortality from all causes (all-cause mortality), nonaccidental mortality, hospital admissions for respiratory illnesses, emergency room (ER) visits for asthma, school loss days; and minor restricted activity days [see Supplemental Material, Table S1 (http://dx.doi.org/10.1289/ehp.1104271) for study details]. For several health effects, two or more C–R functions were pooled (see Supplemental Material, pp. 4–5 for details on pooling and Table S4 for the pooled estimates).

Most of the studies in the air pollution epidemiological literature have estimated exponential (log-linear) C–R functions in which the natural logarithm of the health effect is a linear function of the air pollutant:

*y* = *Be*^β^*^x^,* [1]

where *x* is the ambient air pollutant (e.g., O_3_) level, *y* is the incidence of the health effect at O_3_ level *x*, β is the coefficient of ambient O_3_ concentration, and *B* is the incidence at *x* = 0.

The health impact function—the relationship between a change in the pollutant concentration (Δ*x* = *x_1_*–*x_0_*) and the corresponding change in incidence of the health effect in the population (Δ*y* = *y_1_*–*y_0_*)—derived from the log-linear C–R function is

Δ*y* = *y_0_*[*e*^βΔ^*^x^*–1], [2]

where *x_1_* and *x_0_* represent the model-simulated summertime O_3_ levels ca. 2050 and ca. 2000, respectively, while *y_1_* and *y_0_* represent the health effect incidence in the with and without climate-change (baseline) scenario, respectively. The baseline incidence (*y_0_*) is the product of the baseline incidence rate and the exposed population. The measure of O_3_ concentration available from the climate change–air quality models is the O_3_ season average of the MDA8. The C–R functions relate the MDA8 to health effects, and we applied this O_3_ season average MDA8 to each day. Because the health impact functions are nearly linear, this application of a seasonal average to each day in the season provides a good approximation to the result we would get if we had individual daily 8-hr maxima for each day in the O_3_ season. In many cases, the C–R function used an O_3_ metric other than the MDA8 (e.g., the 24-hr mean) [see Supplemental Material, Table S1 (http://dx.doi.org/10.1289/ehp.1104271)]; the coefficients from these functions were converted to coefficients for the MDA8 (for the methods, see Abt Associates Inc. 2010, Appendix G). This conversion would be expected to add only a small amount of uncertainty to the results.

*Baseline incidence rates.* A detailed description of the estimation of baseline incidence rates ca. 2050 is given in the Supplemental Material, pp. 5–7 (http://dx.doi.org/10.1289/ehp.1104271). Briefly, we calculated cause-specific death counts at the county level for selected age groups from individual-level mortality data for years 2004 through 2006, obtained from the Centers for Disease Control and Prevention (CDC 2008b), National Center for Health Statistics (NCHS), for the entire United States. The county-level death counts were then divided by the corresponding county-level population to obtain the mortality rates. We used 3 years (2004–2006) of mortality and population data to provide more stable estimates. We then extrapolated these county-level mortality rates to 2050 using the U.S. Census Bureau national mortality life tables (U.S. Census Bureau 2010).

Regional rates for hospitalizations and asthma ER visits were calculated from year 1999 regional hospitalizations and year 2000 ER visits obtained from the National Hospital Discharge Survey and the National Hospital Ambulatory Medical Care Survey, respectively (CDC 2008a, 2010) [see Supplemental Material, pp. 6–7 (http://dx.doi.org/10.1289/ehp.1104271)]. We applied the regional rates to every county in a region. Hospitalization rates are cause specific, with causes defined by those combinations of the *International Classification of Diseases, Ninth Revision* (ICD-9) codes (see Supplemental Material, Table S1) that were used in the selected epidemiological studies (e.g., Bell et al. 2004; Ito et al. 2005). However, we were unable to project rates of hospitalizations and ER visits to 2050 because, unlike mortality rates, there are no reliable projections of rates for hospitalizations or for ER visits or for trends into the future.

*Defining the O_3_ season.* The climate change–air quality models used in this analysis generally defined the O_3_ season as June, July, and August (i.e., climatological summer in the Northern Hemisphere). Although most of the air pollution epidemiology studies that have focused on O_3_ have defined the season more broadly (e.g., May through September), we used the more conservative June through August definition for consistency with the O_3_ simulations. Modeling results summarized in Weaver et al. (2009) indicate similar magnitudes of climate-induced O_3_ increases in fall and spring, suggesting that the health impacts we report here are more conservative than if we considered a more standard, longer O_3_ season.

*Estimation of human health impacts*. BenMAP calculated the change in each adverse health effect within each grid cell of the air quality grid by combining the appropriate C–R function coefficient (β), baseline incidence (*y_0_*), and simulated change in O_3_ due to climate change (Δ*x*) in the health impact function (Equation 2). Although BenMAP uses the same “national” C–R function coefficient (β) in all grid cells, population estimates and baseline incidence rates in the health impact function are as location-specific as possible. The grid cell-specific changes in health effects are then summed across grid cells to produce county-level, state-level, and national estimates of health impacts.

## Results

Using the 7 climate change–air quality modeling systems and the 5 population projections, we produced 35 potential answers to the question: How many O_3_-related cases of a given health effect (e.g., nonaccidental mortality) may be attributable to climate change in the conterminous United States in a future year? We also considered more than one C–R function for some health effects, further increasing the number of potential answers.

*National results.* Estimates of the annual national O_3_-related nonaccidental mortality ca. 2050 ranged from > 600 deaths avoided because of climate change to > 2,500 deaths attributable to climate change, depending on the climate change–air quality modeling system, population projection, and C–R function used (Table 3). Estimates for all-cause mortality follow similar patterns according to the climate change–air quality modeling system and population projection [see Supplemental Material, Table S2 (http://dx.doi.org/10.1289/ehp.1104271)]. The broad patterns seen for mortality across the different modeling choices are largely mirrored for the morbidity effect estimates as well, though for some health outcomes the numbers of cases are much larger, for example, in the hundreds of thousands or millions for minor restricted activity days (see Supplemental Material, Tables S3–S7).

**Table 3:** Estimated changes in national summertime (June–August) O_3_-related nonaccidental mortality due to simulated climate change between 2000 and ca. 2050.^a^

Modeling system	Study	Population projection								
		ICLUS-A1	ICLUS-A2	ICLUS-BC	W&P	Census 2000
Illinois-1	Bell et al. 2004	570	520	510	440	170
	Ito et al. 2005	2,560	2,340	2,280	1,970	780
	Schwartz 2005	860	790	770	670	270
Illinois-2	Bell et al. 2004	530	480	480	420	160
	Ito et al. 2005	2,390	2,180	2,160	1,870	710
	Schwartz 2005	810	730	730	640	250
CMU	Bell et al. 2004	480	430	430	350	150
	Ito et al. 2005	2,180	1,950	1,920	1,570	690
	Schwartz 2005	730	660	650	540	240
Harvard	Bell et al. 2004	240	220	230	200	80
	Ito et al. 2005	1,090	1,000	1,030	890	380
	Schwartz 2005	370	340	350	300	130
GNM	Bell et al. 2004	40	30	20	10	–20
	Ito et al. 2005	180	140	80	50	–80
	Schwartz 2005	60	50	30	20	–30
NERL	Bell et al. 2004	10	10	–10	–50	–20
	Ito et al. 2005	50	20	–40	–240	–100
	Schwartz 2005	20	10	–20	–80	–40
WSU	Bell et al. 2004	–150	–140	–110	–60	0
	Ito et al. 2005	–650	–630	–480	–240	0
	Schwartz 2005	–220	–210	–160	–90	0
W&P, Woods & Poole. aNumbers rounded to the nearest 10.												

**Figure 2:**
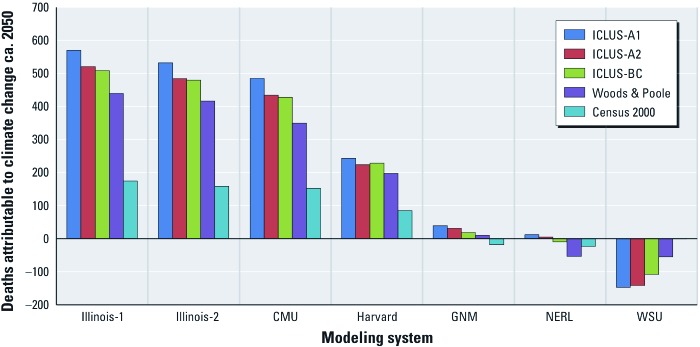
Estimated national summertime (June–August) O_3_-related nonaccidental mortality due to simulated climate change between 2000 and ca. 2050 (C–R function from Bell et al. 2004). We estimated that –0.6 deaths were based on the WSU climate change model–air quality model and Census 2000 population data.

Figure 2 summarizes the influence of the climate change–air quality modeling system and population projection on estimates of future O_3_-related nonaccidental deaths attributable to climate change, using the C–R function described by Bell et al. (2004). The C–R function is itself a source of substantial uncertainty. For example, had we used the C–R function described by Ito et al. (2005) instead, the numbers would have generally been more than 4 times larger (e.g., 2,560 attributable deaths compared with 570 based on Illinois-1 and ICLUS-A1), although the basic pattern according to climate change–air quality modeling system and population projection is the same (Table 3).

Our analysis is one of the first to account for population growth and associated changes in age and geographic distributions. We found that considering these factors has a substantial influence on the estimates of health impacts. The assumption that the population ca. 2050 will be exactly what it was in the year 2000 (i.e., by using Census 2000 population estimates) produces estimates that are consistently lower than those based on population projections, all of which assume at least some increase in population size relative to the year 2000, in addition to changes in the age distribution of future populations, as shown in Figure 2 and Table 3 (and proposed by Tagaris et al. 2009).

The choice of methods to project future age and geographic distributions can also influence results. For example, although the ICLUS-A2 population projection for 2050 is, in total, greater than the ICLUS-A1 projection (424.8 million vs. 386.7 million), ICLUS-A1 is skewed more toward the older age groups [with about 26% projected to be ≥ 65 years of age in 2050 versus only about 21% based on ICLUS-A2; see Supplemental Material, Figure S1 (http://dx.doi.org/10.1289/ehp.1104271)]. Because older people have substantially higher baseline incidence rates for mortality (and other adverse health effects) than do younger people, the same increase in O_3_ concentration would result in more deaths among an older population than among a younger one because the estimated change in the outcome is a function of the baseline incidence, which is the product of the baseline incidence rate and the population size. This is reflected in the slightly higher numbers of O_3_-related deaths for ICLUS-A1, despite the overall smaller population. If C–R functions were available for age group-specific mortality, their application would likely accentuate the importance of age distribution, because older people may be more vulnerable to air pollution.

The importance of the age distribution of the affected population is particularly apparent when we consider morbidity effects that focus on specific age subgroups in the population, such as O_3_-related school days lost (5–17 years of age) or respiratory hospital admissions among those ≥ 65 years of age [see Supplemental Material, Tables S3, S4, S6, S7 (http://dx.doi.org/10.1289/ehp.1104271)]. For example, estimates of O_3_-related respiratory hospital admissions among infants attributable to climate change for a year ca. 2050 based on the ICLUS-A1 population projection are uniformly smaller in magnitude than are the corresponding estimates based on ICLUS-A2 regardless of the climate change–air quality modeling system used (see Supplemental Material, Table S3). This is because ICLUS-A2 projects that a greater percentage of the population (and a larger total population) will be < 1 year of age, and that a smaller percentage of the population will be ≥ 65 years of age, relative to ICLUS-A1 (see Supplemental Material, Figure S1).

Across all of these dimensions, the source of the greatest uncertainty, for both nonaccidental and all-cause mortality, appears to be the projections of future climate change-induced meteorological changes and corresponding air quality changes, which are determined by the climate change–air quality modeling system used. This is shown clearly in the results of an analysis of variance, which decomposes the total variability in estimated mortality into the variability due to the chosen climate change–air quality modeling system, population projection, epidemiological study (C–R function) used, and interactions between these modeling choices, respectively (see Table 4). The different impacts across modeling choices are magnified to a greater or lesser degree by study choice (i.e., by C–R function) [see Supplemental Material, Figure S2 (http://dx.doi.org/10.1289/ehp.1104271)].

**Table 4:** Analysis of variance results for estimates of national summertime (June–August) O_3_-related nonaccidental mortality due to simulated climate change between 2000 and ca. 2050.

Source	df	ANOVA SS	Percent of total SS
Modeling system	6	24,271,499	48
Population projection	4	2,108,558	4
Study	2	9,055,636	18
Modeling system × study	12	10,495,284	21
Modeling system × population projection	24	2,641,882	5
Study × population projection	8	921,745	2
Modeling system × study × population projection	48	1,165,135	2
Total	104	50,659,739	100
Abbreviations: df, degrees of freedom; SS, sum of squares.						

*Regional estimates.* Because national estimates can mask very different regional changes, we delineated three broad regions for additional analysis: the Northeast (defined as east of 100° west longitude and north of 36.5° north latitude); the Southeast (defined as east of 100° west longitude and south of 36.5° north); and the West (defined as everything west of 100° west longitude). These three regions account for the entire continental United States. Finer-scale regional breakdowns, while possible, would have been an overinterpretation of our results given the various uncertainties.

**Figure 3:**
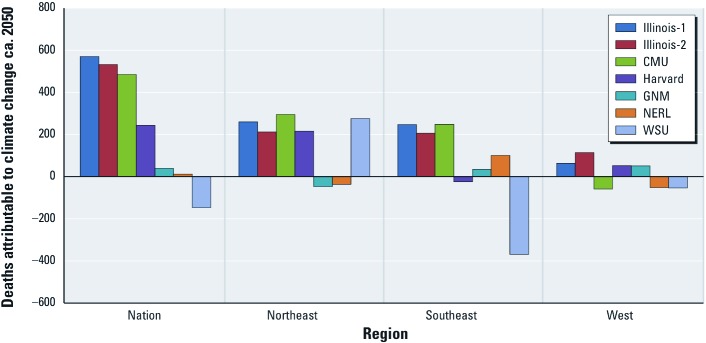
Estimated national and regional summertime (June–August) O_3_-related nonaccidental mortality due to simulated climate change between 2000 and ca. 2050 (C–R function from Bell et al. 2004; ICLUS-A1 population projection).

Figure 3 shows national and regional estimates of O_3_-related nonaccidental mortality using the C–R function from Bell et al. (2004) and the ICLUS-A1 population projection, and it illustrates this national-level masking of differing regional trends. For example, the modest net change in nationwide O_3_-related nonaccidental mortality based on the WSU climate change–air quality modeling system represents the sum of highly variable regional estimates (i.e., 275 avoided deaths in the Northeast, plus 369 additional deaths in the Southeast, plus 54 additional deaths in the West). With the exception of Illinois-1 and Illinois-2, none of the driving climate–air quality scenarios produces regional health impact estimates that are all in the same direction [i.e., increases in the estimated O_3_ concentrations attributable to climate change in some regions are accompanied by decreases in other regions, due, for example, to factors such as differences in circulation patterns and increases in cloud cover (see Weaver et al. 2009)]. Although the WSU climate change–air quality simulation estimates suggest large decreases in O_3_-related deaths in the Southeast and large increases in the Northeast, the GNM and NERL model estimates show regional effects in just the opposite directions. These same general patterns are evident for all-cause mortality and for different C–R functions for either type of mortality.

## Discussion

We have attempted to assess the sensitivity of estimated O_3_-related human health effects of climate change to the following key modeling assumptions and choices: *a*) climate-induced changes in meteorological conditions and the corresponding changes in O_3_ concentrations; *b*) projections regarding the size, and age and geographic distributions of the affected population; and *c*) the C–R relationships linking O_3_ levels to specific health outcomes.

Looking across all combinations of modeling choices (including the climate change–air quality modeling system, population projection, and C–R relationship), estimates of national O_3_-related mortality and morbidity attributable to climate change by mid-century span a wide range (e.g., from roughly 600 cases of nonaccidental mortality avoided as a result of climate change to roughly 2,500 cases attributable to climate change).

The source of the greatest uncertainty at the national level appears to be the climate change–air quality scenario used, with choice of C–R function and population projection also important, though less influential in this analysis. Not only is the total population exposed to O_3_ in a future year important, but assumptions regarding the age distribution of that population are also important for estimating O_3_-related adverse health effects. The variability of these estimates represents the true extent of uncertainty in the problem, however, only to the extent that our choices (seven simulations, five population projections, a few alternative C–R specifications, and a single unchanging set of emissions to air) span the full range of possibilities in their respective dimensions. Thus, our estimates may understate the plausible range of potential future outcomes.

National results can mask important regional differences. Estimates for the Northeast region generally indicated adverse health impacts and were the most consistent across the seven climate–air quality scenarios of the three regions. In contrast, estimated health impacts for the Southeast showed substantial variation. The West generally showed the smallest impacts, largely due to the relatively smaller projected populations.

**Figure 4:**
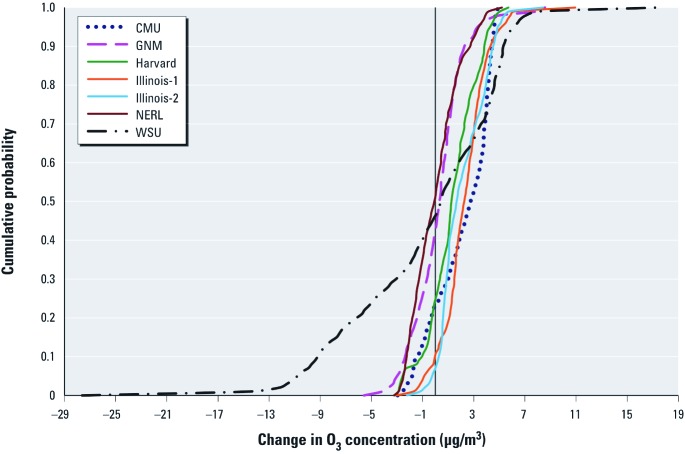
Cumulative probability density functions of national population-weighted summertime O_3_ concentration changes between 2000 and ca. 2050 from the seven sets of climate change–air quality modeling results (ICLUS-A2 population projection; other population projections yielded similar results).

The wide range of estimated O_3_-related mortality and morbidity attributable to climate change resulting from different methodological choices highlights the need to consider an ensemble of estimates, rather than relying on any one modeling system or set of assumptions. Despite this range, however, the large preponderance of the estimates is in the direction of climate-induced increases in O_3_ leading to adverse health impacts. This is illustrated in Figure 4, which shows that population-weighted climate-induced O_3_ concentration changes estimated using the different climate–air quality simulations indicate that 50–90% of the future U.S. population would be subject to increases in O_3_ exposure, all other factors remaining constant.

Finally, as Tagaris et al. (2009) suggested, climate change may have even greater health impacts associated with other air pollutants like PM_2.5_. The combined health effects of O_3_ and these other pollutants, along with other factors such as increased heat waves, should be explored using multipollutant models.

## Conclusion

At this stage in the development of a scientific understanding of climate change and air pollution-related human health, it would be unwise to rely on any one model, epidemiological study, or population projection. This is perhaps the most important message of our analysis. Different combinations of methodological choices and modeling assumptions produce widely varying results, particularly at regional scales, and can produce fundamentally different conclusions about the overall impact of climate change on O_3_-related health effects. The goal of this study was therefore not to develop any best guess as to the most likely future human health impacts of climate-induced O_3_ change, but instead to explore the uncertainty space surrounding assessment of these impacts and to begin to define the envelope of future risk. This also highlights the need to develop decision-making frameworks and tools capable of managing the uncertainty such ensembles represent (e.g., see Johnson and Weaver 2009; Lempert et al. 2004).

## Supplemental Material

(385 KB) PDF
