# Supplementary material for: Variation in Estimated Ozone-Related Health Impacts of Climate Change due to Modeling Choices and Assumptions
Source: Environ Health Perspect. 2012 Jul 12;120(11):1559–64. doi: 10.1289/ehp.1104271 (PMC3556604; doi:10.1289/ehp.1104271)

Supplemental Material

Variation in Estimated Ozone-Related Health Impacts of Climate Change due to Modeling Choices and Assumptions

Authors and Affiliations:
Ellen S. Post[1], Anne Grambsch[2], Chris Weaver[2], Philip Morefield[2], Jin Huang[1], Lai-Yung Leung[3], Christopher G. Nolte[4], Peter Adams[5], Xin-Zhong Liang[6], Jin-Hong Zhu[6], and Hardee Mahoney[1]

[1]Environment and Resources Division, Abt Associates Inc., 4550 Montgomery Ave., Bethesda, MD 20814
[2] Global Change Research Program, National Center for Environmental Assessment, Office of Research and Development, U.S. Environmental Protection Agency, Washington, DC 20460
[3]Pacific Northwest National Labs, Richland, WA 99352
[4] National Exposure Research Laboratory, U.S. Environmental Protection Agency, Athens, GA 30605
[5] Civil & Environmental Engineering, Carnegie Mellon University, Pittsburgh, PA 15213
[6] Department of Atmospheric Sciences, University of Illinois, Urbana, IL 61801

Corresponding Author:
Ellen S. Post
Abt Associates Inc.,

U.S. Mail Delivery Address:
Abt Associates Inc
4550 Montgomery Ave Suite 800
Bethesda, Maryland 20814

Express and Courier Delivery Address:
Abt Associates Inc
4550 Montgomery Ave Suite 800
Bethesda, Maryland 20814

301-347-5287 (phone)
301-828-9662 (fax)
ellen_post@abtassoc.com (email address)

Table of Contents

Adjustment of Air Quality Output from Modeling Systems 3
Extrapolation of 2030 Population Projections by Woods & Poole Economics, Inc. to c. 2050.. 3
Descriptions of Selected ICLUS population projections 4
Pooling of Concentration-Response Functions.. 4
Calculation of Baseline Incidence Rates 5
References.. 8

Tables

Supplemental Material, Table S1. Summary of concentration-response functions used to estimate climate change-related impacts of O_3 on human health 11
Supplemental Material, Table S2. Estimates of national summertime (June-August) O_3-related all-cause mortality due to simulated climate change between 2000 and c. 2050 [a] 12
Supplemental Material, Table S4. Estimates of national summertime (June-August) O_3-related hospital admissions for respiratory illness (ages 65+) due to simulated climate change between 2000 and c. 2050 [a,b] 13
Supplemental Material, Table S5. Estimates of national summertime (June-August) O_3-related emergency room visits for asthma (all ages) due to simulated climate change between 2000 and c. 2050 [a,b].. 14
Supplemental Material, Table S6. Estimates of national summertime (June-August) O_3-related school loss days (ages 5 - 17) due to simulated climate change between 2000 and c. 2050 [a,b] 14
Supplemental Material, Table S7. Estimates of national summertime (June-August) O_3-related minor restricted activity days (ages 18 - 64) due to simulated climate change between 2000 and c. 2050[a,b] 15

Figures

Supplemental Material, Figure S1. Age distributions of ICLUS_A1 and ICLUS_A2 population projections to c. 2050 16
Supplemental Material, Figure S2. Interaction between Model and Study 17

Adjustment of Air Quality Output from Modeling Systems

Benefits analysts who deal with air pollution generally have more confidence in monitored air pollutant concentrations than modeled concentrations, since monitor values are actual measurements. However, unlike modeled values, monitors do not exist in all grid cells of an air quality model grid. Therefore, following EPA's typical procedures for a future-year analysis, we applied a Voronoi Neighbor Averaging (VNA) spatial adjustment to the without-climate-change O_3 metrics, and VNA spatial and temporal adjustments to the with-climate-change O_3 metrics, using both monitor and modeled values in BenMAP. These spatial and temporal adjustment procedures are described in detail in Sections C.3.2 and C.3.3 in Appendix C of Abt Associates Inc. (2010). The change in O_3 due to climate change c. 2050 (Δx in equation (2) in the paper) was then calculated in each of the cells in the 30 km x 30 km grid used for the analysis.

Extrapolation of 2030 Population Projections by Woods & Poole Economics, Inc. to c. 2050

BenMAP uses population growth projections by Woods & Poole Economics, Inc. (Woods & Poole Economics Inc. 2007) to model populations in a future year. Woods & Poole population growth projections incorporate the assumptions from the U.S. Census Bureau population growth model into a comprehensive model of economic and demographic changes over time. These projections are available at the county level for several population sub-groups, defined by age, sex, race, and ethnicity. BenMAP contains a series of population growth projections, based on Woods & Poole data, for each population sub-group in each county. There are 3,109 counties and 304 different population sub-groups per county. (For detailed information about subgroup definitions and forecasting methods, see Section K.1 of the BenMAP User Manual (Abt Associates Inc. 2010).

Woods & Poole population projections are available only through 2030, however, whereas our analysis is c. 2050. Therefore, it was necessary to extrapolate Woods & Poole population projections to 2050. Given the large number of projected population series, we used automatic forecasting algorithms that have been implemented in the forecast package for R (Hyndman 2009; R Development Core Team 2009).

In order to generate our forecasts, we used a set of models that belong to the class of exponential smoothing (ES) forecasting methods (see Gardner 2006 and Hyndman 2009 for the theoretical background of exponential smoothing models.) We evaluated the following three ES models: simple exponential smoothing, linear exponential smoothing, and damped-trend exponential smoothing. These models are categorized by their trend component: none, additive, and damped, respectively. We estimated all three models for each projected population series and then chose the best-fitting model based on the Bayesian Information Criterion (BIC), a standard measure of goodness of fit of a model to the underlying data. The best model was used to forecast each series out to 2050.

These ES forecasting methods try to extrapolate trends seen in a given set of years beyond the final year of the dataset. Thus the set of years on which the extrapolation is based could affect the resulting extrapolation. We applied the method described above to each of the following three series of years: 2000 – 2030; 2010 – 2030; and 2020 – 2030. We then averaged the results. This gives somewhat more weight to the latter years, which is appropriate, since time trends may change over the longer course of years beginning in 2000 or 2010.

The resulting 2050 population forecast was adjusted to match the Census national population projection for 2050 (U.S. Census Bureau 2010a). For each of the 304 population sub-groups we calculated the 2050 national total, as implied by the extrapolated Woods & Poole population projections. We then calculated percent differences between these population totals and the population totals projected by the Census Bureau. Finally, we adjusted each county- and population subgroup-specific extrapolated Woods & Poole projection using corresponding percent differences. This method allowed us to match the Census Bureau national population projection as well as preserve some of the county-specific demographic patterns and trends.

Descriptions of Selected ICLUS population projections

The base case population projection uses the standard Census projection method (U.S. Census Bureau 2010b). A1 represents a world of fast economic development, low population growth, and high global integration. Fertility is assumed to decline and remain low similar to recent and current experience in many European countries (Sardon 2004). The A2 storyline represents a world of continued economic development, but with a more regional focus and slower economic convergence between regions. Fertility is assumed to be higher than in A1. International migration is assumed to be low because a regionally-oriented world would result in more restricted movements across borders. Domestic migration is high because, like in A1, the continued focus on economic development is likely to encourage movement within the United States.

Pooling of Concentration-Response Functions

For several health endpoints, two or more C-R functions were pooled. In particular, for respiratory hospital admissions we undertook the following pooling procedure:

1. Moolgavkar et al. (1997) estimated C-R functions in Minneapolis for hospital admissions (HA), pneumonia (ICD-9 codes 480-487) and HA, COPD (ICD 490-496). We summed the results from these two non-overlapping subcategories.
2. Schwartz (1994b) also estimated C-R functions in Minneapolis for the same two subcategories. However, this study found a significant effect only for HA, pneumonia. So the estimate of “PM-related HA for respiratory illness” in Minneapolis based on Schwartz (1994b) was taken to be just PM-related HA, pneumonia.

3. The estimates of "PM-related HA for respiratory illness" in Minneapolis from (1) and (2) above were pooled using a fixed effects pooling method. (When choosing fixed effects as the pooling method, pooling weights are generated automatically based on the inverse variance of each input result, with the weights normalized to sum to one. Results with a larger absolute variance get smaller weights. For more details, see Section L.2.1.3 in Abt Associates Inc. 2010).
4. Schwartz (1994a) estimated C-R functions for the same two non-overlapping subcategories in Detroit. We similarly summed these results.
5. Finally, Schwartz (1995) estimated C-R functions for "HA, all respiratory" in New Haven, CT and Tacoma, WA. We pooled the HA, All respiratory results from these C-R functions with the results from steps (3) and (4). (For more details, see Section L.2.1.4 in Abt Associates Inc. 2010).

To obtain the asthma ER visits results, we pooled Peel et al. (2005) and Wilson et al. (2005) using the random/fixed effects method (for more detail see Section L.2.1.4 in Abt Associates Inc. 2010). To obtain the results for school absence days, we pooled Gilliland et al. (2001) and Chen et al. (2000) also using the random/fixed effects method.

Calculation of Baseline Incidence Rates

We obtained individual-level mortality data, including residence county FIPS codes,[1] age at death, month of death, and underlying causes (ICD-10 codes), for years 2004-2006 for the entire United States from the Centers for Disease Control (CDC), National Center for Health Statistics (NCHS). The detailed mortality data allowed us to generate cause-specific death counts at the county level for selected age groups. The county-level death counts were then divided by the corresponding county-level population to obtain the mortality rates. To provide more stable estimates, we used three years (2004-2006) of mortality and population data (population data for 2004-2006 were estimates from Woods & Poole Economics, Inc. based on the 2000 Census), i.e.,

$$\textit{Mortality Rate}(2004-2006)_{ijk} = \frac{\sum_{2004}^{2006} death_{ijk}}{\sum_{2004}^{2006} population_{ijk}},$$

where *i* represents the specific cause of mortality (e.g., non-accidental mortality), *j* represents a specific county, and *k* represents a specific age group.

[1] Federal information processing standards codes (FIPS codes) are a standardized set of numeric or alphabetic codes issued by the National Institute of Standards and Technology (NIST) to ensure uniform identification of geographic entities through all federal government agencies. The entities covered include: states and statistically equivalent entities, counties and statistically equivalent entities, named populated and related location entities (such as, places and county subdivisions), and American Indian and Alaska Native areas.

Mortality rates based on 20 or fewer deaths were considered unreliable (see NYSDOH 1999 for an explanation). If the rate for a given cause of death was unreliable in certain counties in a state, we summed up the deaths attributed to that cause in those counties, as well as the populations in those counties and created an aggregate rate for that cause of death in those counties. If that aggregate "state-level" rate was unreliable, we aggregated to the region level (using the four regions defined by the U.S. Bureau of the Census), and if the region-level rate was still unreliable, we aggregated to the national level. At each level of aggregation, only those counties with unreliable rates for the specified cause of death were included. So, for example, if 5 counties in a given state had unreliable rates for a specific cause of death, a "state-level" rate was created by summing the deaths from that cause across those counties and dividing by the sum of the populations in those counties. If this "state-level" rate was still unreliable, we repeated the process at the region level. The aggregate rate estimates were applied only to counties that had "unreliable" data and that estimates for all other counties were based on county-specific estimates.

To project age- and county-specific mortality rates developed using 2004-2006 data to the year 2050, we calculated growth ratios using a series of Census Bureau projected national mortality rates (U.S. Census Bureau 2010a). The procedure we used was as follows:

- For each age group, we calculated the ratio of the Census Bureau national mortality rate projection in year 2050 to the national mortality rate in 2005. Note that the Census Bureau projected mortality rates were derived from crude death rates. The following formula, given by Chiang (1967) (p.2 equation 7), was used: $M = Q/(1-(1-A)*Q)$, where M denotes the projected mortality rate, Q denotes the crude death rate, and A denotes the fraction of the interval (one year) lived by individuals who die in the interval. $A=0.1$ if age < 1, and $A=0.5$ otherwise.

- To estimate mortality rates in 2050 that are both age-group-specific and county-specific, we multiplied age-group-specific mortality rates for 2004-2006 in each county by the appropriate national-level age-group-specific ratios calculated in the previous step. For example, to estimate the projected mortality rate in 2050 among ages 18-24 in Wayne County, MI, we multiplied the mortality rate for ages 18-24 in Wayne County in 2004-2006 by the ratio of Census Bureau projected national mortality rate in 2050 for ages 18-24 to Census Bureau national mortality rate in 2005 for ages 18-24.

Note that future mortality rates are projected to decrease over time.

Hospitalizations

Regional hospitalization counts were obtained from the National Center for Health Statistics' (NCHS) National Hospital Discharge Survey (NHDS) (CDC 2008). NHDS is a sample-based survey of non-Federal, short-stay hospitals (<30 days), and is the principal source of nationwide hospitalization data. Note that the following hospital types are

excluded from the survey: hospitals with an average patient length of stay of greater than 30 days, federal, military, Department of Veterans Affairs hospitals, institutional hospitals (e.g. prisons), and hospitals with fewer than six beds. The survey collects data on patient characteristics, diagnoses, and medical procedures. Public use data files for the year 1999 survey were downloaded and processed to estimate hospitalization counts by region (CDC 2010a). NCHS groups states into four regions using the following groupings defined by the U.S. Census Bureau (2001):

- **Northeast** - Maine, New Hampshire, Vermont, Massachusetts, Rhode Island, Connecticut, New York, New Jersey, Pennsylvania
- **Midwest** - Ohio, Indiana, Illinois, Michigan, Wisconsin, Minnesota, Iowa, Missouri, North Dakota, South Dakota, Nebraska, Kansas
- **South** - Delaware, Maryland, District of Columbia, Virginia, West Virginia, North Carolina, South Carolina, Georgia, Florida, Kentucky, Tennessee, Alabama, Mississippi, Arkansas, Louisiana, Oklahoma, Texas
- **West** - Montana, Idaho, Wyoming, Colorado, New Mexico, Arizona, Utah, Nevada, Washington, Oregon, California, Alaska, Hawaii

We used the 2000 Census of Population and Housing to obtain more age specificity, and then corrected the 2000 Census figures so that the total population equaled the total for 1999 forecasted by NHDS. In particular, for each type of hospital admission (ICD code or codes) we: (1) calculated the count of hospital admissions by region in 1999 for the age groups of interest, (2) calculated the 2000 regional populations corresponding to these age groups, (3) calculated regional correction factors that equal the regional total population in 1999 divided by the regional total population in 2000, (4) multiplied the 2000 population estimates by these correction factors, (5) divided the 1999 regional count of hospital admissions by the estimated 1999 population, and (6) applied the regional rates to every county in that region.

Like mortality rates, the hospitalization rates are cause-specific and the hospital admissions endpoints are defined by different combinations of ICD codes that are used in the selected epidemiological studies.

Emergency Room Visits for Asthma

Regional counts of asthma-related emergency room visit counts were obtained from the National Hospital Ambulatory Medical Care Survey (NHAMCS) (CDC 2010b). NHAMCS is a sample-based survey, conducted by NCHS. The target universe of the NHAMCS is in-person visits made in the United States to emergency and outpatient departments of non-Federal, short-stay hospitals (hospitals with an average stay of less than 30 days) or those whose specialty is general (medical or surgical) or children's general. Public use data files for the year 2000 survey were downloaded and processed to estimate hospitalization counts by region (CDC 2010c). We obtained population estimates from the 2000 Census of Population and Housing. The NCHS regional groupings described above were used to estimate regional emergency room visit rates.

References

Abt Associates Inc. 2010. BenMAP: Environmental Benefits Mapping and Analysis Program, User's Manual. Bethesda, MD: Abt Associates Inc. Available: http://www.epa.gov/air/benmap/docs.html [accessed 14 October 2010].

Bell ML, McDermott A, Zeger SL, Samet JM, Dominici F. 2004. Ozone and short-term mortality in 95 US urban communities, 1987-2000. JAMA 292(19):2372-2378.

Bell ML, Dominici F, Samet JM. 2005. A meta-analysis of time-series studies of ozone and mortality with comparison to the national morbidity, mortality, and air pollution study. Epidemiology 16(4):436-445.

Burnett, RT; Smith-Doiron, M; Stieb, D; Raizenne, ME; Brook, JR; Dales, RE; et al. (2001) Association between ozone and hospitalization for acute respiratory diseases in children less than 2 years of age. Am J Epidemiol 153(5):444-452

CDC (Centers for Disease Control and Prevention). 2008. National Hospital Discharge Survey. Hyattsville, MD:Centers for Disease Control and Prevention. Available: http://www.cdc.gov/nchs/nhds/nhds_questionnaires.htm [accessed 12 October 2010].

CDC (Centers for Disease Control and Prevention). 2010a. National Hospital Discharge Survey. Hyattsville, MD:Centers for Disease Control and Prevention. Available: ftp://ftp.cdc.gov/pub/Health_Statistics/NCHS/Datasets/NHDS/ [accessed 12 October 2010].

CDC (Centers for Disease Control and Prevention). 2010b. National Hospital Ambulatory Medical Care Survey. Hyattsville, MD:Centers for Disease Control and Prevention. Available: http://www.cdc.gov/nchs/ahcd/ahcd_questionnaires.htm [accessed 12 October 2010].

CDC (Centers for Disease Control and Prevention). 2010c. National Hospital Ambulatory Medical Care Survey. Hyattsville, MD:Centers for Disease Control and Prevention. Available: ftp://ftp.cdc.gov/pub/Health_Statistics/NCHS/Datasets/NHAMCS/ [accessed 12 October 2010].

Chen L, Jennison BL, Yang W, Omaye ST. 2000. Elementary school absenteeism and air pollution. Inhal Toxicol 12(11):997-1016.

Chiang PL. 1967. Variance and covariance of life table functions estimated from a sample of deaths. National Center for Health Statistics. Washington, DC. March 1967.

Gardner Jr. ES. 2006. Exponential smoothing: The state of the art — Part II. Int J Forecasting 22:637-666.

Gilliland FD, Berhane K, Rappaport EB, Thomas DC, Avol E, Gauderman WJ, et al. 2001. The effects of ambient air pollution on school absenteeism due to respiratory illnesses. Epidemiology 12(1):43-54.

Hyndman R. 2009. Forecast: Forecasting Functions for Time Series, R Package Version 1.24. Available: http://robjhyndman.com/software/forecast/ [accessed 29 September 2010].

Ito K, De Leon SF, Lippmann M. 2005. Associations between ozone and daily mortality: analysis and meta-analysis. Epidemiology 16(4):446-457.

Levy JI, Chemerynski SM, Sarnat JA. 2005. Ozone exposure and mortality: an empiric bayes metaregression analysis. Epidemiology 16(4):458-468.

Moolgavkar SH, Luebeck EG, Anderson EL. 1997. Air pollution and hospital admissions for respiratory causes in Minneapolis St. Paul and Birmingham. Epidemiology 8(4):364-370.

NYSDOH (New York State Department of Health). 1999. Rates based on small numbers: statistics teaching tools. Available: http://www.health.state.ny.us/diseases/chronic/ratesmall.htm [accessed 13 October 2010].

Ostro BD, Rothschild S. 1989. Air Pollution and Acute Respiratory Morbidity—an Observational Study of Multiple Pollutants. Environ Res 50(2):238-247.

Peel JL, Tolbert PE, Klein M, Metzger KB, Flanders WD, Todd K, et al. 2005. Ambient air pollution and respiratory emergency department visits. Epidemiology 16(2):164-174.

R Development Core Team. (2009) R: A Language and Environment for Statistical Computing. R Foundation for Statistical Computing, Vienna, Austria. Available: http://www.R-project.org [accessed 29 September 2010].

Sardon JP, Robertson DA. 2004. Recent demographic trends in the developed countries. Population 59(2):263-314.

Schwartz J. 1994a. Air pollution and hospital admissions for the elderly in Detroit, Michigan. Am J Respir Crit Care Med 150(3):648-655.

Schwartz J. 1994b. PM(10) ozone, and hospital admissions for the elderly in Minneapolis St Paul, Minnesota. Arch Environ Health 49(5):366-374.

Schwartz J. 1995. Short term fluctuations in air pollution and hospital admissions of the elderly for respiratory disease. Thorax 50(5):531-538.

U.S. Census Bureau. 2001. Census 2000 Geographic Definitions. Available: http://www.census.gov/geo/www/geo_defn.html [accessed 13 October 2010].

U.S. Census Bureau. 2010a. U.S. Population Projections. Available: http://www.census.gov/population/www/projections/downloadablefiles.html [accessed 12 October 2010].

U.S. Census Bureau. 2010b. U.S. Population Projections. Available: http://www.census.gov/population/www/projections/natdet-D5.html [accessed 12 October 2010].

US EPA (United States Environmental Protection Agency). 2009. Assessment of the Impacts of Global Change on Regional U.S. Air Quality: A Synthesis of Climate Change Impacts on Ground-Level Ozone An Interim Report of the U.S. EPA Global Change Research Program.

Wilson AM, Wake CP, Kelly T, et al. 2005. Air pollution, weather, and respiratory emergency room visits in two northern New England cities: an ecological time-series study. Environ Res 97(3):312-321.

Woods & Poole Economics Inc. 2007. Complete Demographic Database. Washington, DC:Woods & Poole Economics Inc. Available: http://www.woodsandpoole.com/index.php [accessed 12 October 2010].

Supplemental Material, Table S1. Summary of concentration-response functions used to estimate climate change-related impacts of O_3 on human health

Health Endpoint	Study	Location	Age Range	Metric	Beta	Std. Err.	Notes
Mortality, All Cause	Bell et al. (2005)	US & non-US cities	All ages	Daily 8-hour max. [1]	0.000795	0.000212	Warm season
Mortality, All Cause	Levy et al. (2005)	US & non-US cities	All ages	Daily 8-hour max. [2]	0.001119	0.000179	Warm season
Mortality, Non-Accidental	Bell et al. (2004)	95 US cities	All ages	Daily 8-hour max. [1]	0.000261	0.000089	Warm season
Mortality, Non-Accidental	Ito et al. (2005)	Meta-analysis [7]	All ages	Daily 8-hour max. [1]	0.001173	0.000239	Warm season
		Meta-analysis	All ages	Daily 8-hour max. [2]	0.000532	0.000088	
Hospital admission (HA), All Respiratory	Burnett et al. (2001)	Toronto, CAN	0-1	Daily 8-hour max. [2]	0.008177	0.002377	Warm season
HA , COPD [4]	Moolgavkar et al. (1997)	Minneapolis, MN	65+	Daily 8-hour max. [1]	0.00196	0.001238	All year
HA , Pneumonia [4]	Moolgavkar et al. (1997)	Minneapolis, MN	65+	Daily 8-hour max. [1]	0.00266	0.000762	All year
HA , Pneumonia [4]	Schwartz (1994a)	Minneapolis, MN	65+	Daily 8-hour max. [1]	0.002784	0.001305	All year
HA , COPD (less asthma) [4]	Schwartz (1994b)	Detroit, MI	65+	Daily 8-hour max. [1]	0.003424	0.001293	All year
HA , Pneumonia [4]	Schwartz (1994b)	Detroit, MI	65+	Daily 8-hour max. [1]	0.003230	0.000806	All year
HA , All respiratory [4]	Schwartz (1995)	New Haven, CT	65+	Daily 8-hour max. [1]	0.001777	0.000936	Warm season
HA , All Respiratory [4]	Schwartz (1995)	Tacoma, WA	65+	Daily 8-hour max. [1]	0.004931	0.001770	Warm season
ER, Asthma[5]	Peel et al. (2005)	Atlanta, GA	All ages	Daily 8-hour max.	0.000870	0.000529	
ER, Asthma[5]	Wilson et al. (2005)	Portland, ME	All ages	Daily 8-hour max.	0.003000	0.001000	
ER, Asthma[5]	Wilson et al. (2005)	Manchester, NH	All ages	Daily 8-hour max.	-0.001000	0.002000	
School Loss Days, All Cause[6]	Chen et al. (2000)	Wachoe Co, NV	5-17	Daily 8-hour max. [2]	0.015763	0.004985	All year
School Loss Days, All Cause[6]	Gilliland et al. (2001)	Southern California	5-17	Daily 8-hour max.[3]	0.007824	0.004445	All year
Minor Restricted Activity Days		Nationwide	18-64	Daily 8-hour max. [2]	0.002596	0.000776	

[1] Converted from 24-hour mean.
[2] Converted from daily 1-hour maximum
[3] Converted from 8-hour mean
[4] These studies were pooled in BenMAP to generate pooled incidence estimates for respiratory hospital admissions.
[5] These studies were pooled in BenMAP to generate pooled incidence estimates for asthma-related ER visits. Note: Jaffe et al. (2003) is listed in Table 6-2 of EPA's O_3 NAAQS RIA as being among those studies included in the pooled analysis for asthma-related ER visits. However, we were informed via personal communication with Neal Fann (EPA/OAQPS) that this study was ultimately not included because it covered a substantially different age range (ages 5 – 34) from the other studies.
[6] These studies were pooled in BenMAP to generate pooled incidence estimates for school loss days.
[7] [illegible]

Supplemental Material, Table S2. Estimates of national summertime (June-August) O_3-related all-cause mortality due to simulated climate change between 2000 and c. 2050 [a]

Climate Change/Air Quality Model	Study	Population Projection				
		ICLUS_A1	ICLUS_A2	ICLUS_BC	Woods & Poole	Census_2000
Illinois-1	Bell et al. (2005)	1810	1660	1620	1410	570
	Levy et al. (2005)	2550	2340	2280	1990	810
Illinois-2	Bell et al. (2005)	1690	1540	1530	1340	520
	Levy et al. (2005)	2380	2180	2150	1890	730
CMU	Bell et al. (2005)	1530	1380	1360	1120	500
	Levy et al. (2005)	2160	1940	1910	1570	700
Harvard	Bell et al. (2005)	770	710	730	630	280
	Levy et al. (2005)	1090	1000	1020	890	390
GNM	Bell et al. (2005)	120	100	60	30	-60
	Levy et al. (2005)	170	140	80	40	-80
NERL	Bell et al. (2005)	40	20	-30	-170	-80
	Levy et al. (2005)	50	20	-40	-240	-110
WSU	Bell et al. (2005)	-470	-450	-350	-180	-10
	Levy et al. (2005)	-660	-640	-490	-260	-10

[a] Rounded to the nearest 10.

Supplemental Material, Table S3. Estimates of national summertime (June-August) O_3-related hospital admissions for respiratory illness (ages <1) due to simulated climate change between 2000 and c. 2050[a,b]

Climate Change/Air Quality Model	Population Projection				
	ICLUS_A1	ICLUS_A2	ICLUS_BC	Woods & Poole	Census_2000
Illinois-1	1570	2650	1990	2350	1600
Illinois-2	1610	2740	2060	2350	1610
CMU	1250	2060	1550	1830	1290
Harvard	710	1230	940	1100	820
GNM	190	310	200	170	10
NERL	-40	-100	-100	-100	-160
WSU	-430	-770	-540	-510	-190

[a] Rounded to the nearest 10.
[b] Because of the lack of reliable projections of hospitalization rates, the numbers in the table were based on current rather than projected future baseline incidence rates.

Supplemental Material, Table S4. Estimates of national summertime (June-August) O_3-related hospital admissions for respiratory illness (ages 65+) due to simulated climate change between 2000 and c. 2050 [a,b]

Climate Change/Air Quality Model	Population Projection				
	ICLUS_A1	ICLUS_A2	ICLUS_BC	Woods & Poole	Census_2000
Illinois-1	6050	5500	5410	4850	1940
Illinois-2	5650	5120	5110	4630	1780
CMU	5190	4630	4580	3880	1670
Harvard	2530	2320	2410	2130	940
GNM	300	220	80	10	-250
NERL	70	10	-140	-620	-310
WSU	-1480	-1420	-1050	-650	30

[a] Rounded to the nearest 10.
[b] Because of the lack of reliable projections of hospitalization rates, the numbers in the table were based on current rather than projected future baseline incidence rates.

Supplemental Material, Table S5. Estimates of national summertime (June-August) O_3-related emergency room visits for asthma (all ages) due to simulated climate change between 2000 and c. 2050 [a,b]

Climate Change/Air Quality Model	Population Projection				
	ICLUS_A1	ICLUS_A2	ICLUS_BC	Woods & Poole	Census_2000
Illinois-1	1370	1710	1490	1760	1290
Illinois-2	1330	1670	1460	1720	1240
CMU	1230	1500	1300	1490	1130
Harvard	700	870	770	900	730
GNM	-80	-130	-130	-180	-220
NERL	-90	-130	-130	-170	-200
WSU	0	-60	0	-60	190

[a] Rounded to the nearest 10.
[b] Because of the lack of reliable projections of ER visit rates, the numbers in the table were based on current rather than projected future baseline incidence rates.

Supplemental Material, Table S6. Estimates of national summertime (June-August) O_3-related school loss days (ages 5 - 17) due to simulated climate change between 2000 and c. 2050 [a,b]

Climate Change/Air Quality Model	Population Projection				
	ICLUS_A1	ICLUS_A2	ICLUS_BC	Woods & Poole	Census_2000
Illinois-1	633000	925000	743000	880000	659000
Illinois-2	638000	937000	755000	893000	650000
CMU	522000	745000	599000	679000	545000
Harvard	299000	445000	362000	422000	347000
GNM	50000	67000	44000	35000	-29000
NERL	-25000	-50000	-50000	-67000	-84000
WSU	-134000	-212000	-153000	-197000	-27000

[a] Rounded to the nearest 1000.
[b] Based on current rather than projected future baseline incidence rates.

Supplemental Material, Table S7. Estimates of national summertime (June-August) O_3-related minor restricted activity days (ages 18 - 64) due to simulated climate change between 2000 and c. 2050[a,b]

Climate Change/Air Quality Model	Population Projection				
	ICLUS_A1	ICLUS_A2	ICLUS_BC	Woods & Poole	Census_2000
Illinois-1	1959000	2063000	1934000	2333000	1681000
Illinois-2	1941000	2049000	1927000	2362000	1612000
CMU	1637000	1688000	1582000	1818000	1436000
Harvard	926000	990000	941000	1131000	872000
GNM	120000	108000	73000	58000	-78000
NERL	-76000	-109000	-130000	-202000	-213000
WSU	-333000	-375000	-301000	-460000	2000

[a] Rounded to the nearest 1000.

[b] Based on current rather than projected future baseline incidence rates.

Supplemental Material, Figure S1. Age distributions of ICLUS_A1 and ICLUS_A2 population projections to c. 2050

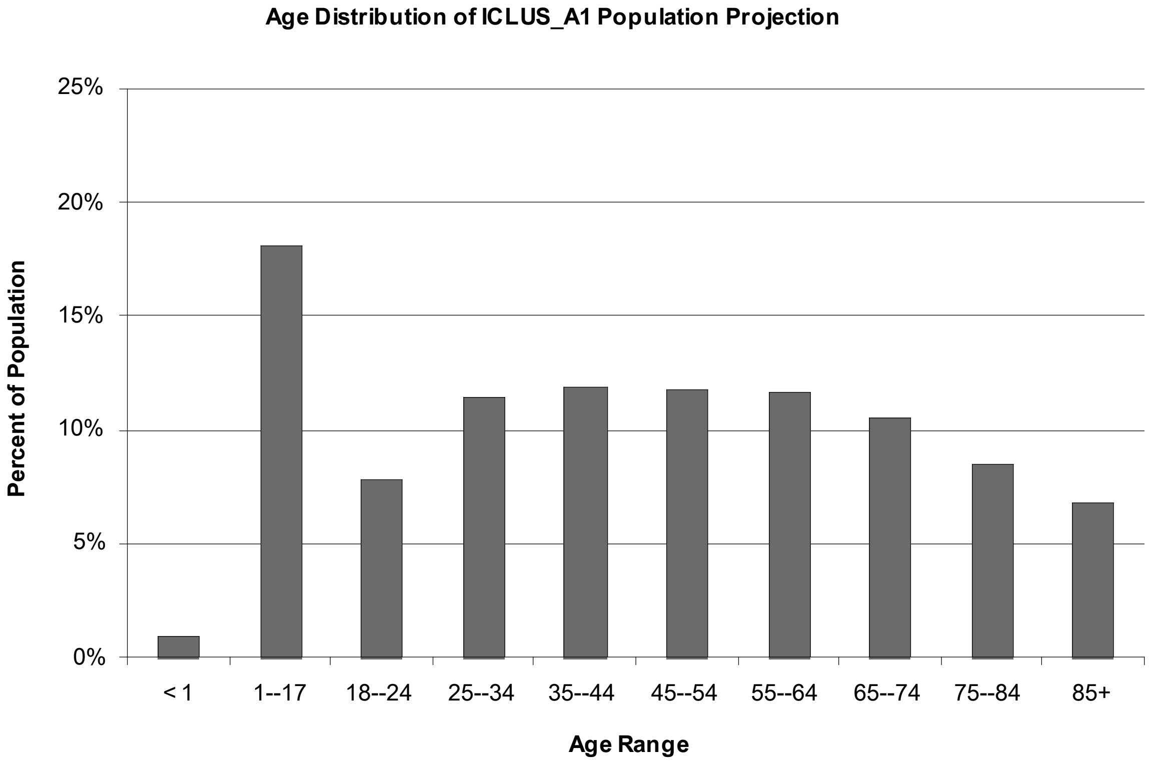

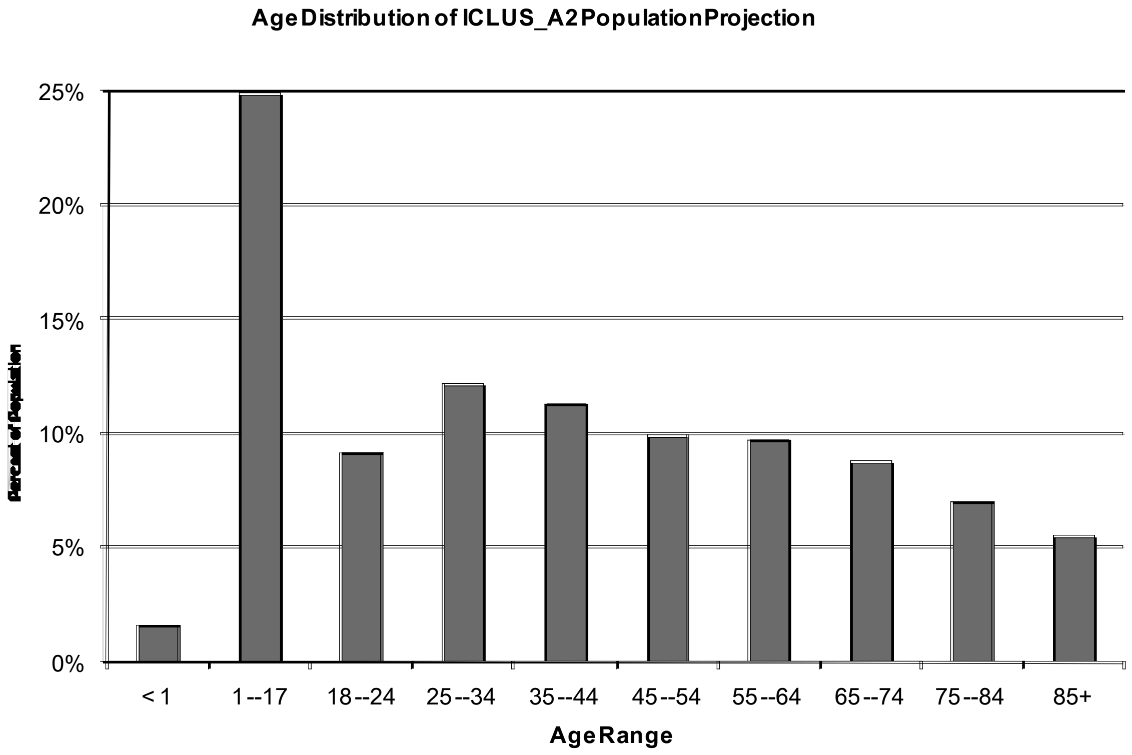

Supplemental Material, Figure S2. Interaction between Model and Study

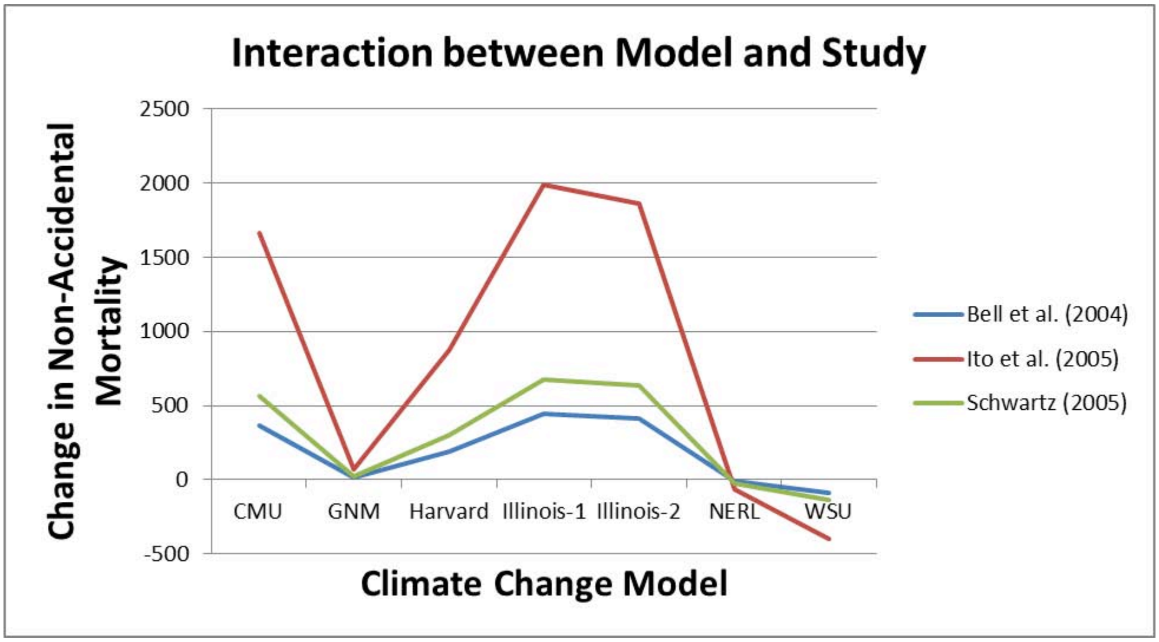

Supplement: (385 KB) PDF [file ehp.1104271.s001.pdf]
